# Analysis of health care professionals’ incident reports on medical devices in Croatia

**DOI:** 10.3325/cmj.2023.64.265

**Published:** 2023-08

**Authors:** Antonela Šimunović, Krunoslav Kranjčec, Marija Pekas, Siniša Tomić

**Affiliations:** Croatian Agency for Medicinal Products and Medical Devices, Zagreb, Croatia

## Abstract

**Aim:**

To assess the quantity and quality of incident reports on medical devices by health care professionals from 2012 to 2021 and evaluate the effect of reporting on manufacturers’ post-market surveillance.

**Methods:**

Eighty-five incident reports were scored according to a self-developed evaluation system, and categorized as excellent, good, medium, qualified, and unqualified. The completeness of data in critical fields was assessed. For each report, the type and city of the reporter, and medical device risk class were extracted to calculate the frequency of report occurrence per risk class and outcomes for reportable reports.

**Results:**

The number of reports received from health care professionals was low; the highest number of reports in a year was 17. The majority of reports were deemed as unqualified (61.18%) and only 4.71% as excellent. Still, 67.65% of incident reports importantly affected the manufacturer’s post-market surveillance, either as added information that contributes to risk monitoring or directly triggering a field safety corrective action.

**Conclusion:**

The number of total reports and reports per year shows extensive underreporting in Croatia, and the quality of the provided reports is insufficient.

The medical device market consists of over 500 000 products divided into 10 000 generic groups (1). Medical devices facilitate diagnosis and treatment, but their use is associated with risks, especially when it comes to new technologies (2). One of the mechanisms that ensures patients’ safety is incident reporting (IR). The purpose of IR is to identify safety issues regarding medical device usage. IR may lead to an investigation and interventions to reduce harm to patients (3,4). Adapted from high-risk industries, it is set up with the aim of learning from incidents rather than pinpointing the culprit (5). Healthcare professionals should report incidents to the manufacturer or the national competent authority, which then forward the report to the manufacturer without delay (6). Reporting by health care professionals in Croatia is legally binding, but there is no penalty for failing to do so (7).

Even though most health care professionals are aware of the IR system, only a fraction decides to file reports (8). Incident reporting is low among physicians compared with nurses (9). The low number of reports hinders the main goal of vigilance systems, making it difficult to identify risks, enable learning, and prioritize patients’ safety (10,11).

There have been good examples of improving medical devices after serious safety incidents. However, despite the widespread establishment of medical device vigilance, major medical device failures and serious health impact for patients have been revealed (12). The IR system is still perceived as a source of confusion for health care professionals (12,13). This study aimed to assess the reporting behavior of Croatian health care professionals from 2012 to 2021 and the impact of reports on the manufacturer’s post-market surveillance and patients’ safety.

## METHODS

### Study design

We reviewed all health care professionals’ incident reports (N = 85) received by email from 2012 to 2021 on a designated form accessible on the website of the Croatian Agency for Medicinal Products and Medical Devices (HALMED). All the reports submitted in any form other than the designated incident report form were translated into the corresponding form before the assessment.

### Evaluation criteria

Two HALMED assessors with relevant experience in vigilance reports processing reviewed all the fields in designated incident reports to establish which fields were considered relevant for proper report assessment. Fields in the designated incident report form were divided into two groups: those relevant for processing and those that can be omitted from the study. The fields relevant for processing were further assessed and ranked based on their content relevance. The fields containing information of higher relevance, specifically the information crucial for risk evaluation and for further processing of the incident were assigned two points (Table 1). All other fields were assigned one point. The maximum number of points per report was 64.

**Table 1:** Incident report fields assigned two points and the rate of their completion

Report field		Rate (%)
Reporter information	Name	89.41
	E-mail	73.53
Manufacturer information	Name	77.06
	E-mail	23.53
	Country	31.18
Distributer information	Name	39.16
	E-mail	27.11
Medical device information	Commercial name/brand name/make	94.12
	Serial or lot/batch number(s) (if applicable)	47.65
Incident information	Date the incident occurred	91.76
	Incident description narrative	99.41
	Medical device current location/disposition (if known)	33.33
Patient information	Patient outcome	85.71

### Report evaluation

After evaluation criteria were defined, the two assessors analyzed the reports and evaluated the information provided in the fields. If a consensus was reached that the required information was complete, the field was assigned a maximum number of points (one or two points depending on the relevance of that field). When the information was only partly complete, the assessors would decide on the appropriate number of points depending on the information provided. If a field contained information that was not usually expected for that field or if the field was lacking information completely, it was assigned zero points. Also, any misplaced information was considered lacking. The total number of points for all fields was summarized for each report, expressed as percentages in relation to the maximum number of possible points per report, and categorized. The report quality was categorized according to an evaluation system developed for the purposes of this study. The five levels of classification (total score = 100%) were as follows: excellent -≥90; good - 80-89; medium - 70-79; qualified - 60-69; unqualified <60 points.

The scores were also used to assess the reports’ average quality per year. The assessors determined in which fields the provided information was most and least complete. For each report, the type and city of the reporter and medical device risk class were extracted to calculate the frequency of report occurrence per risk class. Also, the outcomes of reportable incident reports were determined to assess their impact on the manufacturer’s post-market surveillance. Any event that meets the following three basic reporting criteria is considered as an incident and must be reported to the relevant competent authorities: an event has occurred, the manufacturer’s device is suspected to be a contributory cause, and the event led or might have led to death or serious deterioration in the state of health of a patient, user, or another person (6).

## RESULTS

The study involved 85 health care professionals’ incident reports received by HALMED between 2012 and 2021. The maximum number of reports per year was 17 (Table 2). Most of the reports came from Zagreb (30.59%), followed by Split and Osijek (both 10.59%), and most came from hospitals (67.9%), followed by pharmacies.

**Table 2:** Number of reports per year

Year	Number of reports
2012	3
2013	2
2014	10
2015	5
2016	17
2017	14
2018	12
2019	10
2020	7
2021	5

**Figure 1:**
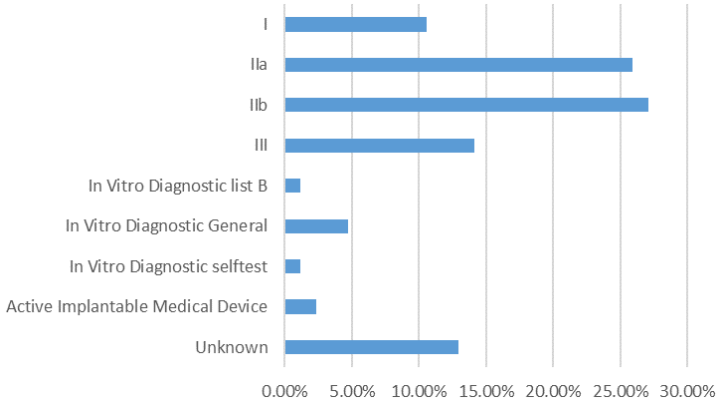
The percentage of each medical device risk class in the received reports.

Only 37 out of 85 reporters provided the general categorization for the medical device involved in the incident report. For every report where the risk class was not supplied and insufficient information was provided to identify the class risk, we assigned it in order to assess the risk class reporting frequency (Figure 1). The two most reported medical device risk classes were IIa and IIb, which cover a wide range of invasive and non-invasive devices used in hospitals and sold in pharmacies. Risk classes are assigned to medical devices according to rules set out in Annex VIII of the new EU Regulations (14,15).

In this study, the quality of the report refers to whether the form’s fields were filled in with the requested information. In total, four reports (4.71%) were deemed excellent, while 52 (61.18%) were deemed unqualified (Table 3).

**Table 3:** Number of reports per category

Category	Number of reports	Percentage
Excellent	4	4.71
Good	14	16.47
Medium	9	10.59
Qualified	6	7.06
Unqualified	52	61.18

The only fields that reached the scores over 90% were “Commercial name/brand name/make,” “Date the incident occurred,” and “Incident description narrative.” The critical field that was completed most frequently in the initial report was “Incident description narrative” (99.41%), and the least frequently completed field was “Manufacturer e-mail” (23.53%) (Table 1).

**Figure 2:**
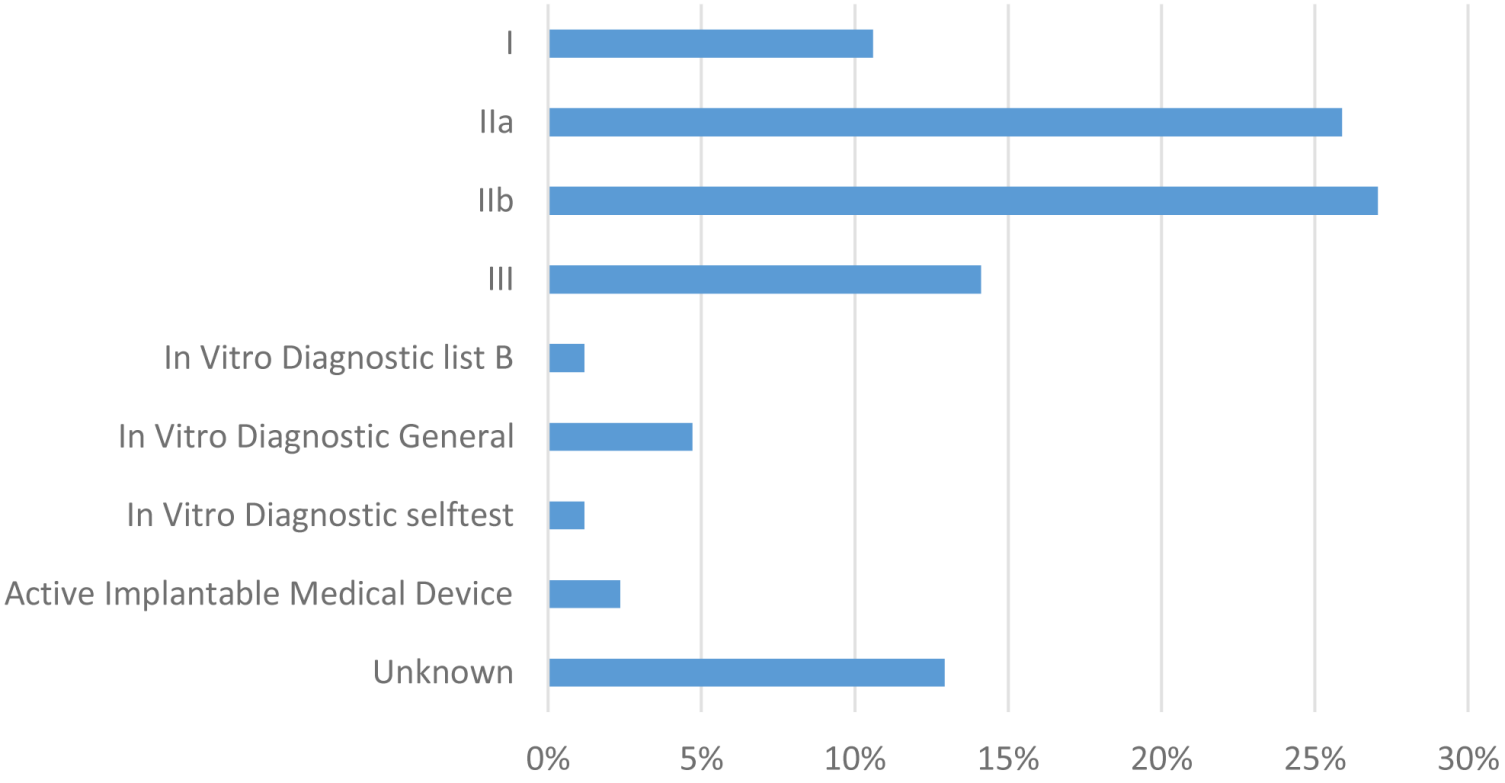
Outcomes for reportable incident reports. FSCA - field safety corrective action.

In 14.71% of reportable incidents, the manufacturer’s investigation directly led to an initiation of a field safety corrective action, which means that collectively 52.94% of incident reports affected the manufacturer’s post-market surveillance, either as added information that contributes to the process of risk monitoring or directly triggering the initiation of FSCA (Figure 2).

## DISSCUSSION

In this study, both the quantity and quality of health care professionals’ incident reports were poor. In regards to the overall number of the received reports, the effect of the reports on manufacturers’ post-market surveillance was limited. However, health care professionals still play a crucial role in medical device vigilance.

The number of received incident reports was concerningly low and unrealistic for the size of the Croatian market. In 2019, there were 715 639 hospitalized patients with an average length of hospital stay of 8.17 days (16). It is hard to imagine that with so many patients and provided medical procedures, there occurred only 10 incidents related to medical devices. The number of reports per year clearly indicates an extensive underreporting practice in Croatia.

The quality of the reports was also insufficient. The low scores can be partly explained by the fact that a large number of the received reports (45.88%) were on forms designated for a medicinal product, which do not contain the same information fields as the designated incident form. The designated incident form for health care professionals is a shortened version of the Manufacturer Incident Report form used by manufacturers to report to competent authorities. Due to simplification of the form and the fact that the risk class for medical device is mostly available only on the manufacturer’s EU declaration of conformity, health care professionals are usually not aware of the risk class for medical device. For that reason, the section “Medical device risk class” on the designated incident form consists of only three basic groups: medical device, active medical device, and *in vitro* medical device. Despite the simplification, only 37 out of 85 reporters provided the general categorization for the medical device involved in the incident report. The low quality of reports may negatively affect the processing of incident reports, ultimately obstructing further investigation and interventions. After the report is forwarded to the manufacturer, a common practice is that low-quality incident reports are deemed non-reportable due to missing information. Another consequence is a prolonged processing of the incident and a delayed corrective action. An additional problem is that reporters often do not respond to HALMED’s or manufacturers’ attempts to obtain more information (Figure 2).

Only 34 out of 85 reports were assessed as reportable incidents, meaning that they fulfilled all three criteria for incidents to be reported (6). This finding indicates that reporters do not have sufficient knowledge of which incidents need to be reported. In 38.24% of reportable incidents, the threshold limit value for the number of expected incidents for that particular medical device was not exceeded, thus prompting the manufacturer only to add new information to the device’s risk file and closely monitor future similar incidents. Collectively, 52.94% of incident reports affected the manufacturer’s post-market surveillance, either as added information that contributes to the process of risk monitoring or directly triggering the initiation of FSCA. Even with such a small sample of incident reports, we see that health care professionals have a crucial role in vigilance systems, and their reports may bring about important positive changes in patients’ care.

Low scores in some critical fields can mostly be explained by reporting on forms not designated for medical device incident reporting, such as the form for medicinal products. All fields that scored below 50% are not present on the other submitted form, except the field “Serial or lot/batch number(s),” which scored 47.65%. The information on serial or lot/batch numbers is important for the manufacturer’s investigation, because when this information is not present in the incident report, the manufacturer will often dismiss the case without further investigation deeming it impossible. Another critical field that strongly affects the manufacturer’s investigation but is not provided on other submitted forms is “Medical device current location/disposition,” since the manufacturer is usually interested in collecting the device involved in the incident to perform an investigation. Unfortunately, health care professionals often do not track the whereabouts of the medical device involved in the incident. In 68.67% of the reports, health care professionals were unaware of medical device location and in 7.23% the device was discarded.

Another concerning observation was that the field “User facility report reference number” (not assessed as critical) was completed in only 4 out of 85 reports. This might indicate that the majority of health care institutions in Croatia do not have established vigilance recording systems and therefore cannot generate reference numbers.

In comparison with health care professionals, distributors who make a device available on the market up until the point of putting it into service (other than the manufacturer or the importer) (14,15) submitted only four incident reports in the same period. Such a low number of incident reports may be explained by manufacturer-distributor business relation. It will be intriguing to see if the number of reports will change as new EU Regulations, for the first time, oblige distributors to inform the competent authority in their country that the device presents a serious risk (14,15).

Other studies also showed poor quality of incident reports submitted by health care professionals. The main reasons for lack of engagement, particularly by doctors, are lack of time, a feeling that nothing will improve, poor safety culture in the organization, and a lack of training (1,17,18). Often, the incidents are not reported due to the fear of identifying user error in planning or executing a medical procedure (19). Feedback following IR in health care is essential for learning from failures and for maintaining health care professionals’ motivation (20). Another study (21) showed that health care professionals usually do not report a safety problem if the problem can be fixed. Such a way of thinking does not contribute to the patients’ safety or to prevention of the same type of problems (21).

The purpose of reporting is to trigger an investigation that will further provide the details of the incident. Thus, incident reports do not need to be elaborate but they need to contain enough information to trigger an investigation (12).

A path forward to increasing patients’ safety is to change the reporting culture and encourage active user reporting while at the same time not focusing on blame and personal responsibility. Higher reporting rates are associated with well-established safety culture (3) and teamwork perceptions (22). Healthcare professionals’ reporting reflects the organizational transparency and the drive to ensure patient safety and quality improvement (23). The most reliable health care institutions are those that prioritize monitoring of patients’ safety (24). Similar measures for increasing reporting rates are proposed by most authors: developing a more user- friendly and efficient IR system, providing feedback on the results arising from reports, having a professional in charge of reporting within institutions, and education of health care professionals (2,17,18,25). It is also worth mentioning the drawbacks of incident reporting as a method: reports are sometimes too subjective and unfair, not comparable between health care institutions, and carry unacknowledged bias (23).

The study has some limitations. A low number of received reports limits a more in-depth analysis of the information received in IR. A larger sample of incident reports could give us a clearer picture of reporting practices in Croatia and on the reports’ effect on patients’ safety. The analysis was further impaired by the fact that almost half of the reports were submitted on a wrong form. This study is also limited in its relevance and generalization to other settings and populations.

In conclusion, the study showed poor reporting practices by health care professionals in Croatia. Both the number of reports and their quality indicate insufficient user engagement in the vigilance system. The number of incident reports could be increased through continuous education of health care professionals and by simplifying the reporting process.
